# Association of prenatal alcohol exposure and prenatal depressive symptoms with offspring hair cortisol in childhood and adolescence

**DOI:** 10.1186/s12888-025-07559-9

**Published:** 2025-11-05

**Authors:** Anne-Christine Plank, Kerstin Panaseth-Gehle, Jennifer Gerlach, Stefan Mestermann, Peter A. Fasching, Matthias W. Beckmann, Oliver Kratz, Gunther H. Moll, Bernd Lenz, Johannes Kornhuber, Anna Eichler, IMAC-Mind Konsortium

**Affiliations:** 1 Department of Child and Adolescent Mental Health, University Hospital Erlangen, Friedrich-Alexander-Universität Erlangen-Nürnberg, 91054 Erlangen, Germany; 2 Department of Obstetrics and Gynecology, University Hospital Erlangen, Friedrich-Alexander-Universität Erlangen-Nürnberg, 91054 Erlangen, Germany; 3 Department of Addictive Behavior and Addiction Medicine, Central Institute of Mental Health (CIMH), Medical Faculty Mannheim, Heidelberg University, 68159 Mannheim, Germany; 4 Department of Psychiatry and Psychotherapy, University Hospital Erlangen, Friedrich-Alexander-Universität Erlangen-Nürnberg, 91054 Erlangen, Germany

**Keywords:** Prenatal alcohol exposure (PAE), Meconium ethyl glucuronide (EtG), Maternal self-report, Prenatal depression, Hair cortisol, Pregnancy, Childhood, Adolescence

## Abstract

**Background:**

Prenatal alcohol exposure (PAE) and maternal depressive symptoms are associated with hypothalamic-pituitary-adrenal (HPA) axis alterations in the offspring. The present study investigated long-term associations of both risk factors on the offspring’s hair cortisol concentration (HCC) in childhood and adolescence.

**Methods:**

The HCC of *n* = 94 children was assessed at primary school age (T1, *M* = 7.7 years, *SD* = 0.81) and in early adolescence (T2, *M* = 13.3 years, *SD* = 0.30). PAE was operationalized by maternal self-report and the meconium alcohol metabolite ethyl glucuronide (EtG), applying two cut-off values, EtG ≥ 10 ng/g (EtG_10_+: *n *= 18) and EtG ≥ 154 ng/g (EtG_154_+: *n* = 9). The Edinburgh Postnatal Depression Scale (EPDS) was used to screen for prenatal maternal depressive symptoms (EPDS ≥ 10: *n* = 24). The clinical relevance of the results was assessed by correlating the HCC with children’s emotional and behavioral problems as measured by the Strengths and Difficulties Questionnaire (SDQ).

**Results:**

The EtG_10_+ and the EtG_154_+ group showed lower HCC at T1 than the respective control groups, with a significant difference observed for the EtG_154_ risk group (*p* = .032). This difference was attenuated at T2. Children and adolescents whose mothers reported prenatal depressive symptoms did not show any significant differences in HCC at any time.

**Conclusion:**

The present study provides further evidence of long-term effects of alcohol consumption during pregnancy on the HPA axis development of the child, as manifested in distinct trajectories of HCC from primary school age to early adolescence.

**Clinical trial number:**

Not applicable.

## Background

The development of a child and its susceptibility to diseases later in life is influenced by various exogenous and endogenous factors that act both pre- and postnatally. One of the most harmful prenatal risk factors is alcohol consumption during pregnancy [1]. However, alcohol remains the most commonly used substance among women of reproductive age worldwide [2], with a prevalence of around 25% in Europe [3]. Prenatal alcohol exposure (PAE) has multiple toxic effects during embryo-fetal development and causes fetal alcohol spectrum disorders (FASD), which comprise a variety of long-term structural abnormalities, neurocognitive disturbances and behavioral impairments [4]. The latter often manifest in attention deficit hyperactivity disorder (ADHD) symptoms, including hyperactivity, impulsivity, poor attention, and slower reaction times [5]. The majority of clinical studies indicate that even low doses of alcohol and/or alcohol consumption early in pregnancy can negatively impact the child’s development and later mental health [6, 7].

In addition to PAE, a mother’s mental health during pregnancy and the first year of her child’s life is considered an influential factor in the cognitive, social, and emotional development of her child. According to a meta-analysis by Woody et al. [8], 9.2% of women in higher-income countries and 19.2% in low- and middle-income countries experience prenatal depression, which is one of the earliest risk factors for fetal and child development [9]. Prenatal depressive symptoms are associated with negative birth outcomes and health effects in childhood [10, 11]. Numerous studies have reported a higher risk for emotional and behavioral abnormalities in affected offspring, including externalizing and internalizing problems, negative affect and impaired mental health [10, 12].

The exact mechanisms behind the negative developmental consequences of PAE and depressive symptoms are not yet fully understood [13]. However, a significant amount of evidence suggests that an altered activity of the child’s hypothalamic-pituitary-adrenal (HPA) axis is a potential mediator of such developmental outcomes. The HPA axis is essential for maintaining homeostasis and responding to stress via cortisol release. During puberty, the HPA axis undergoes significant developmental changes, with adolescents showing greater HPA axis activity and higher overall cortisol levels compared to younger children [14]. HPA axis activity and recovery is regulated by a complex interplay of neural and hormonal processes, and alterations in HPA axis activity have been associated with several somatic, psychosomatic and psychiatric disorders [15]. For instance, depression and internalizing symptoms have been linked to elevated basal cortisol activity and reactivity in both youths and adults [16–18]. In contrast, research indicates that externalizing problems are associated with reduced basal cortisol activity in primary school aged children [19] and with a blunted cortisol response to stress in adolescents [20]. During fetal development and early childhood, the HPA axis undergoes significant maturation, making it sensitive to the potentially maladaptive influence of stressors [21, 22]. Several preclinical and clinical studies provide evidence of an association between PAE and HPA axis alterations in the offspring, which comprise a dysregulated basal cortisol activity, most commonly manifested as hypercortisolism, and an increased reactivity to stress [22–24]. Similarly, research suggests a link between prenatal depressive symptoms in mothers and changes in the HPA axis in their children. However, the outcomes of these studies vary: They report both an increased [25, 26] and a reduced basal cortisol secretion [27, 28] as well as a U-shaped relationship between prenatal depressive symptoms and cortisol reactivity [29] or no significant correlation at all [30, 31].

Previous studies investigating the association between PAE or prenatal depressive symptoms and changes in the child’s cortisol stress system have primarily focused on the short-term activity of the HPA axis by measuring basal or stress-dependent cortisol concentrations in saliva or blood (e.g., [26–28, 32, 33]). In contrast, fewer studies have examined the effects of prenatal depression on the offspring’s HPA axis by evaluating cumulative hair cortisol concentrations (HCC) in children or adolescents (e.g., [31, 34, 35]). Regarding PAE, we have previously shown PAE-associated alterations in both salivary and cumulative hair cortisol in children at primary school age [36]. The present study aimed to longitudinally extend these findings by investigating whether there is a long-term association between PAE (assessed by maternal self-report and the alcohol metabolite ethyl glucuronide (EtG) in meconium) and changes in the offspring’s HPA axis activity (measured by HCC) in childhood and adolescence. Additionally, we examined the long-term influence of prenatal maternal depressive symptoms on the offspring’s HPA axis activity. To assess the functional relevance of our findings, we also tested whether there were correlations between HCC and behavioral problems in children and adolescents with PAE / prenatal maternal depressive symptoms.

## Materials and methods

### Study design

The present study is based on data derived from the prospective longitudinal FRAMES (Franconian Maternal Health Evaluation Studies) / FRANCES (Franconian Cognition and Emotion Studies) cohort study [5, 37]. For FRAMES (2005–2007), *n* = 1100 women of legal age (≥ 18 years) and in the 3rd trimester (≥ gestational week 30) of an intact pregnancy were recruited by the Department of Obstetrics and Gynecology, Erlangen, Germany. Data were collected pre-, peri- and postnatally (during the 3rd trimester, 48–72 h after delivery and 6–8 months after birth) and comprised interview- and questionnaire-based information about the women’s mental health and substance use during pregnancy. Birth outcomes of their children were registered immediately after delivery, and a newborn meconium sample was collected within the first 24 h after birth (T0).

Between 2012 and 2015, when children attended primary school, a subsample of these women (*n* = 618) was contacted for re-participation. Finally, *n* = 245 FRAMES mother-child dyads (39.6%; child age: *M* = 7.74, *SD* = 0.74, range 6.00–9.90) agreed to take part in the FRANCES I (T1) follow-up wave. Mothers with an existing prenatal risk, in terms of prenatal depressive symptoms and/or alcohol consumption, were contacted more actively: in addition to an invitation letter, which was sent to all families, families with a prenatal risk who did not respond to the first invitation were contacted by phone. This recruiting process of FRANCES resulted in a targeted risk oversampling (higher prevalence of depressive symptoms/alcohol exposure in FRANCES [26.4/22.7%%] than FRAMES [17.4/17.9%]). The *n* = 245 participating women did not differ from the 373 non-participating women in marital status (*χ*^*2*^(1) = 0.16, *p* = .690), educational level (*χ*^*2*^(1) = 0.08, *p* = .774) or family income (*χ*^*2*^(2) = 0.97, *p* = .616) at time of childbirth (FRAMES data). Mothers and children took part in two data collection sessions in person (91.4%) or only filled out questionnaires by post (8.60%).

The mothers and children, who took part in FRANCES I, were contacted again from 2019 to 2021, during early adolescence, to take part in the second follow-up wave: FRANCES II (T2). 186 (75.9%) of the 245 contacted families (with *n* = 188 children, due to two pairs of twins) agreed to participate again (child age: *M* = 13.3, *SD* = 0.34, range 12.8–14.5), of which 167 families (89.8%) participated in person and 21 families (10.2%) only filled out questionnaires by post. A total of 32 families (13.1%) did not want to continue the participation, another 27 families (11.0%) were not accessible anymore. When comparing participating families with non-participating families, no differences in marital status (*χ*^*2*^(1) = 0.35, *p* = .552), family income (*χ*^*2*^(4) = 3.94, *p* = .414) or maternal total psychopathology (*t*(234) = -0.93, *p* = .353) at time of FRANCES I were found. However, higher educated mothers were more often willing to re-participate (*χ*^*2*^(1) = 7.60, *p* = .006).

The study was approved by the Ethics Committee of the Friedrich-Alexander-University Erlangen-Nürnberg (FAU) (4596 and 353_18B) and conducted in accordance with the Declaration of Helsinki. Prior to the commencement of the study, all parents and children/adolescents gave informed consent/assent.

### Sample characteristics

For present data analyses, valid T1 and T2 HCC values of *n* = 117 children who participated and provided hair samples in both FRANCES I and FRANCES II were available. Data from twins (*n* = 4) were excluded, as their intrauterine environment is not comparable to singleton pregnancies. Further exclusion criteria were: missing EtG and EPDS value (*n* = 1), acute or chronic disease (*n* = 1), smoking during pregnancy (*n* = 0), medication intake within the last 6 months prior to sampling (corticosteroids: *n* = 16; ketoconazole: *n* = 4; somatropin: *n* = 1) and chemical hair treatment within the last 30 days prior to sampling (*n* = 1) at T1 and/or T2. After exclusion of outliers (|*z*| >3.29, *n* = 3), the final sample comprised *n* = 94 participants.

### Measures and materials

#### Prenatal risk variables

##### Prenatal alcohol exposure (PAE)

To assess PAE during the 3rd trimester of pregnancy, the concentration of the ethanol metabolite EtG was measured in the meconium of the newborn, in which EtG accumulates from week 20 of gestation until birth. One gram of meconium was collected within the first 24 h after birth and stored at − 80 °C until analysis. EtG was analyzed via mass spectrometry as described by Bakdash et al. and Goecke et al. [37, 38]. The recognized limit of detection for EtG is 10 ng/g [38]; however, the value to classify PAE varies from study to study. Based on published research, two different cut-off-values were applied: EtG = 10 ng/g [5, 36], and EtG = 154 ng/g [5] as correlate of more pronounced alcohol exposure.

##### Maternal self-report on prenatal alcohol consumption

In the 3rd trimester of their pregnancy (T0), the mothers were asked in a structured face-to-face interview about their drinking behavior during pregnancy [37]. Based on their answer to the question “Did you drink alcohol during your current pregnancy?“, they were assigned to one of two groups for data analysis: No drinking: “I don’t drink in general” / “I didn’t drink during pregnancy”; drinking: “I rarely drank during pregnancy” / “I drank one glass/day during pregnancy” / “I drank more than one glass/day during pregnancy”.

##### Depressive symptoms during pregnancy

The German version of the Edinburgh Postnatal Depression Scale (EPDS) [39], which is also suitable for assessing prenatal depression [40], was used to determine depressive symptoms during pregnancy. In a German study with 110 women, an EPDS threshold of 9.5 had a sensitivity of 0.96, a specificity of 1 and a positive predictive value of 1. The EPDS analysis gave a Guttman split-half reliability value of 0.82 and an α coefficient value of 0.81 [41]. In their 3rd trimester (T0), women were asked in 10 items for symptoms during the last seven days on a four-point rating scale (0 = lowest depressive symptomatology to 3 = highest depressive symptomatology). A total score ranging from 0 to 30 was calculated and women with a score ≥ 10 were considered to show depressive symptoms.

#### Hair cortisol concentration (HCC)

To determine the cumulative cortisol level of the month before sampling, a strand of hair was cut as close as possible at the posterior vertex. The first proximal centimeter was used for analysis, as hair grows approximately 1 cm per month. The samples were individually wrapped in tin foil and stored at + 4 °C until further analysis. The average storage time was *M* = 19.50 weeks (*SD* = 11.12) at T1 and *M* = 22.41 weeks (SD = 10.28) at T2. Mothers were asked about medication intake and chemical hair treatments of their children.

##### Cortisol / protein extraction

Cortisol and protein were extracted from samples collected at T1 as described by Grimm et al. [36]. Briefly, samples were incubated with 2 mL methanol for 24 h, the methanol supernatant was transferred into a fresh tube and evaporated at 60 °C overnight to obtain a dry, methanol-free pellet. This pellet was then dissolved in phosphate-buffered saline (PBS) and stored at 4 °C until analysis. To the samples collected at T2, an improved extraction method was applied as described by Frisch et al. [42]. Briefly, the samples were washed with isopropanol (2×), air dried at room temperature, weighed and grinded in a ball mill (Retsch GmbH, Haan, Germany). The following 4-step extraction method comprised an incubation step with 1 mL of methanol for 24 h, followed by centrifugation (10,000 × *g*, 2 min) and separation of the supernatant, a second incubation step with 1 mL of acetone for 5 min (again followed by centrifugation and separation of the supernatant), and one repetition of both steps. Finally, the pooled methanol-acetone supernatant of each sample was evaporated at 50 °C. The resulting pellet was stored at -20 °C until analysis and dissolved in 250 µl PBS immediately before analysis.

##### Determination of protein concentrations / HCC

Protein concentrations were determined via Bradford Protein Assay (Roti-Quant Protein quantitation assay according to Bradford, Carl Roth GmbH, Germany) to be used for normalization of HCC values at T1 (see below) due to technical reasons. Cortisol concentrations were measured using salivary cortisol ELISA kits (T1: KA1885, Abnova, Taipei, Taiwan; T2: RE52611; IBL International, Hamburg, Germany) according to the manufacturers’ instructions. The intra- and inter-assay coefficients of variation (CV) were < 10%. Each sample was assayed in duplicate (Benchmark Plus microplate spectrophotometer, Bio-Rad Laboratories, Hercules, CA, USA) and the mean values and CV were computed for each duplicate.

##### HCC data processing

The final HCC values were calculated as follows: Cortisol concentrations were normalized to the respective sample protein concentration (T1) / sample weight (T2) and ln-transformed (since standardized HCC were not normally distributed) (see also Frisch et al. [42]):

$$\eqalign{ &{\rm{HCC}}\>{\rm{T1:}}\>{\rm{Cortisol - to - protein}}\> \cr & {\rm{(HCC/P)}}\>{\rm{ratio}}\>{\rm{(pg/mg)}}\>{\rm{ = }}\>{\rm{ln}}\left( {{{{\rm{HCC}}\>\left( {{{{\rm{ng}}} \over {{\rm{ml}}}}} \right)} \over {{\rm{HPC}}\>\left( {{{\mu \>{\rm{g}}} \over {ml}}} \right)}}\>{\rm{x}}\>{{10}^6}} \right) \cr} $$

$$\eqalign{& {\rm{HCC}}\>{\rm{T2:}}\>{\rm{Cortisol - to - weight}} \cr & {\rm{(HCC/W)}}\>{\rm{ratio}}\>{\rm{(pg/mg)}} \cr & {\rm{ = }}\>{\rm{ln}}\left( {{{{\rm{HCC}}\>\left( {{{{\rm{ng}}} \over {{\rm{ml}}}}} \right){\rm{x}}\>0.25\>{\rm{ml}}} \over {{\rm{hair}}\>{\rm{sample}}\>{\rm{weight}}\>\left( {{\rm{mg}}} \right)}}\>{\rm{x}}\>1000} \right) \cr} $$

In addition, the ln-transformed HCC/P and HCC/W ratios were z-standardized for better comparability of T1 and T2 values.

#### Emotional and behavioral problems (children)

In order to investigate the associations between PAE / prenatal depressive symptoms, HCC and children’s emotional and behavioral problems, psychopathological symptoms of the child were rated by the mother at T1 and T2 via the German version of the Strengths and Difficulties Questionnaire (SDQ) [43, 44]. The SDQ includes 25 items to be rated on a 3-scaled Likert scale. The items are divided into five-item subscales (ranges 0–10): Emotional symptoms, Conduct problems, Hyperactivity/inattention problems, Peer relationship problems, and Prosocial behavior. Studies confirm that the five-factor structure is largely replicated in the German versions [44]. In a German population sample of *n* >900, the mother-reported scores yielded a Cronbach’s alpha of between 0.82 for the total score and 0.52 for conduct and 0.66 for emotional problems [45]. SDQ parents scores distinguished clinical from general populations and correlated well with CBCL scores (Child Behavior Checklist) [46]. In the present study, the Total Difficulties score and, in accordance with the internalizing vs. externalizing disease spectrum, the subscales “Emotional symptoms” and “Conduct problems” were calculated.

#### Potential confounders

The following variables were considered as potential confounders: sex assigned at birth, age, body-mass-index [BMI, calculated from weight and height measured in FRANCES II (kg/m^2^) and presented as percentiles accounting for sex and age [47], use of antibiotics during the past 6 months (see [36]), hair sampling in winter season (Dec-Feb), birth weight, APGAR Score [average of the three APGAR scores recorded in the maternity room (1, 5, and 10 min after birth), rating the infant’s heart rate, breathing efforts, reflexes, muscle tone and color; maximum score: 10], socioeconomic status [SES, calculated on the basis of parental education (4: 12/13 years of schooling; 3: 10 years of schooling; 2: 9 years of schooling; 1: < 9 years of schooling) and family income (6 levels: < 1000 Euro/month to >5000 Euro/month), sum index theoretical range: 3–14], maternal psychopathology [assessed using the German version of the Brief Symptom Inventory (BSI) [48] and calculating the Global Severity Index (GSI), see Gerlach et al. [49] for further details].

### Statistical analyses

Data were analyzed using IBM^®^ SPSS^®^ Statistics (Version 28.0) (Armonk, NY: IBM Corp, 2021). Prenatal risk variables were dichotomized as follows: Meconium EtG: 1.: EtG cut-off 10 ng/g (EtG_10_): EtG_10_+ = EtG-values ≥ 10 ng/g and EtG_10_− = EtG-values < 10 ng/g. 2.: EtG cut-off 154 ng/g (EtG_154_): EtG_154_+ = EtG-values ≥ 154 ng/g; EtG_154_− = EtG-values < 154 ng/g. Results of self-reports on alcohol consumption during pregnancy were grouped in self-report− (= women declaring no alcohol consumption during pregnancy) and self-report+ (= women declaring alcohol consumption during pregnancy). Depressive symptoms during pregnancy were recorded as EPDS ≥ 10, vs. EPDS < 10. Descriptive data are presented as frequencies (*n*), mean values (*M*) and standard deviations (*SD*). The sizes of the analyzed samples vary due to missing data (incomplete information from either mother or offspring) regarding some sample characteristics; varying sample sizes are indicated in the notes of the tables.

For all analyses, the probability of error was α = 5% as the level of significance was defined as *p* < .05 (two-tailed). First, differences between each prenatal risk group and the respective control group (EtG_10_+ vs. EtG_10_−, EtG_154_+ vs. EtG_154_−, self-report + vs. self-report−, EPDS ≥ 10 vs. EPDS < 10) regarding sample characteristics were analyzed using *t* tests and *χ*^*2*^ tests. Levene’s test was used to test for the assumption of homogeneity of variance, and degrees of freedom (*df*) were adjusted if the assumption was not met. Associations between HCC/P (T1) or HCC/W (T2) *z*-scores and potential covariates were analyzed via Pearson correlations or *t* tests. Repeated measures analyses of covariance (ANCOVA) were performed to assess group differences in HCC over time [within-subject factor ‘time’: HCC T1 and HCC T2], with separate ANCOVAs calculated for each risk/control group [between-subject factor ‘group’: (1) EtG_10_ (EtG_10_+ and EtG_10_−), (2) EtG_154_ (EtG_154_+ and EtG_154_−), (3) self-report (self-report+ and self-report−) and (4) EPDS (EPDS ≥ 10 and EPDS < 10)]. Variables that were significantly (*p* < .05) correlated with HCC/P and/or HCC/W or which significantly differed between risk group-positive and -negative children were controlled for in ANCOVA analyses. For each ANCOVA, homogeneity of variance was asserted using Levene’s tests. When significant interaction effects were identified in the ANCOVAs, pairwise comparisons were made using *post-hoc* tests [Least Significant Difference (LSD)]. In case of significant HCC differences between the risk and control groups, the functional relevance of the findings within the risk groups was assessed using confounder controlled partial correlations of HCC with the SDQ scales and total SDQ score. Results are presented as *t* values, *χ*^*2*^ values, *r* and *F* values. Effect sizes are reported as Cohen’s *d* / *φ* / *r* and interpreted according to Cohen [50] as small (0.20 ≤ |*d*| ≤ 0.49 / 0.10 ≤ |*r* or *φ*| ≤ 0.29), medium (0.50 ≤ |*d*| ≤ 0.79 / 0.30 ≤ |*r* or *φ*| ≤ 0.49) or large (|*d*| >0.79 / |*r* or *φ*| >0.49). For ANCOVA effect size estimations, partial eta squared (*η*_*p*_^*2*^) is reported (*η*_*p*_^*2*^ < 0.06: small effect, 0.06 ≤ *η*_*p*_^*2*^ ≤ 0.14: medium effect, *η*_*p*_^*2*^ >0.14: large effect).

**Table 1:** Sample characteristics and mean/frequency comparison for the risk factor PAE

Parameters	Total *n* = 94	EtG (cutoff 10 ng/g) *n* = 81					EtG (cutoff 154 ng/g) *n* = 81					Self-report *n* = 94				
		EtG_10_− *n* = 63	EtG_10_+ *n* = 18				EtG_154_− *n* = 72	EtG_154_+ *n* = 9				Self-report− *n* = 71	Self-report+ *n* = 23			
	M (SD)	M (SD)	M (SD)	t (df)	*p*	d	M (SD)	M (SD)	t (df)	*p*	d	M (SD)	M (SD)	t (df)	*p*	d
**HCC/P T1 (pg/mg)**	164819.45 (498689.9)	106392.66 (147427.13)	56908.72 (88055.19)				103643.36 (143610.1)	29419.22 (25693.58)				113235.94 (159479.33)	324055.48 (967091,54)			
Ln-transformed	10.97 (1.32)	10.88 (1.19)	10.33 (1.132)				10.87 (1.17)	9.87 (1.06)				10.92 (1.20)	11.10 (1.66)			
Ln-transformed and z-standardized	0.022 (0.967)	−0.040 (0.873)	−0.446 (0.83)	1.76 (79)	0.082	0.47	−0.049 (0.857)	−0.782 (0.777)	2.44 (79)	0.017*	0.86	−0.010 (0.881)	0.122 (1.21)	−0.57 (92)	0.570	−0.14
**HCC/W T2 (pg/mg)**	2.26 (1.52)	2.199 (1.64)	2.198 (1.2)				2.16 (1.59)	2.52 (1.19)				2.34 (1.60)	2.04 (1.27)			
Ln-transformed	0.603 (0.682)	0.538 (0.734)	0.644 (0.557)				0.529 (0.717)	0.827 (0.462)				0.632 (0.684)	0.515 (0.683)			
Ln-transformed and z-standardized	−0.004 (0.921)	−0.092 (0.992)	0.051 (0.753)	−0.57 (79)	0.574	−0.15	−0.105 (0.968)	0.299 (0.625)	−1.22 (79)	0.228	−0.43	0.035 (0.924)	−0.124 (0.923)	0.715 (92)	0.476	0.17
**Birth outcomes**																
Birth weight (grams)	3486.01 (490.73)	3459.13 (430.06)	3643.06 (531.69)	−1.52 (79)	0.133	−0.41	3464.31 (457.05)	3785.56 (368.99)	−2.02 (79)	0.046	−0.72	3444.44 (490.74)	3614.35 (478.48)	−1.45 (92)	0.150	−0.35
APGAR score	9.43 (0.56)	9.47 (0.44)	9.33 (0.75)	0.73 (19.10)	0.474	0.27	9.46 (0.53)	9.33 (0.47)	0.62 (77)	0.53	0.23	9.49 (0.43)	9.25 (0.82)	1.37 (26.18)	0.182	0.44
**Primary school age (T1)**																
Age (years)	7.74 (0.81)	7.49 (0.60)	7.72 (0.67)	−1.39 (79)	0.169	−0.37	7.52 (0.63)	7.72 (0.44)	−0.90 (79)	0.373	−0.32	7.60 (0.72)	8.16 (0.93)	−2.63 (31.01)	0.013*	−0.72
BMI (percentile)	51.90 (23.41)	51.10 (22.78)	63.50 (18.29)	−2.12 (79)	0.037*	−0.57	52.44 (22.05)	65.11 (22.93)	−1.62 (79)	0.110	−0.57	50.56 (24.92)	56.04 (17.81)	−1.15 (52.18)	0.254	−0.23
SES index	11.35 (2.18)	11.17 (2.28)	11.83 (1.98)	−1.11 (79)	0.269	−0.30	11.31 (2.21)	11.44 (2.40)	−0.176 (79)	0.861	−0.06	11.32 (2.23)	11.43 (2.06)	−0.21 (92)	0.834	−0.05
Maternal psycho- pathology	46.52 (13.77)	45.37 (12.63)	51.39 (16.42)	−1.44 (23.16)	0.164	−0.44	45.23 (13.15)	58.56 (12.76)	−2.87 (78)	0.005**	−1.02	44.93 (13.33)	51.64 (14.25)	−2.03 (91)	0.045	−0.50
**Adolescence (T2)**																
Age (years)	13.28 (0.30)	13.25 (0.24)	13.31 (0.39)	−0.644 (20.96)	0.527	−0.22	13.25 (0.26)	13.40 (0.43)	−1.02 (8.72)	0.336	−0.53	13.25 (0.27)	13.36 (0.36)	−1.53 (92)	0.129	−0.37
BMI (percentile)	50.44 (29.83)	49.61 (28.54)	62.39 (31.44)	−1.63 (78)	0.106	−0.44	51.10 (29.19)	63.44 (31.49)	−1.19 (78)	0.239	−0.42	47.84 (29.15)	58.35 (31.15)	−1.48 (91)	0.144	−0.35
SES index	11.91 (2.03)	11.78 (2.11)	12.24 (1.92)	−0.81 (73)	0.424	−0.22	11.93 (2.03)	11.50 (2.45)	0.55 (73)	0.586	0.21	11.92 (2.02)	11.86 (2.10)	0.12 (85)	0.906	0.03
Maternal psycho- pathology	45.89 (11.69)	45.65 (11.53)	44.06 (11.33)	0.517 (78)	0.607	0.14	44.96 (11.30)	47.89 (12.85)	−0.72 (78)	0.472	−0.26	46.39 (12.16)	44.19 (10.03)	0.76 (90)	0.451	0.19
	***n***	***n***	***n***	***Χ***^***2***^ ***(df)***	***p***	***φ***	***n***	***n***	***Χ***^***2***^ ***(df)***	***p***	***φ***	***n***	***n***	***Χ***^***2***^ ***(df)***	***p***	***φ***
Sex assigned at birth (female/male)	48/46	32/31	10/8	0.13 (1)	0.721	0.04	37/35	5/4	0.06 (1)	0.814	0.03	37/34	11/12	0.13 (1)	0.721	−0.04
**Primary school age (T1)**																
Antibiotics (yes/no)	16/78	14/49	1/17	2.58 (1)	0.108	−0.18	15/57	0/9	2.30 (1)	0.129	−0.17	12/59	4/19	0.00 (1)	0.957	0.01
Hair sampling in winter (yes/no)	34/60	24/39	6/12	0.14 (1)	0.712	−0.04	27/45	3/6	0.06 (1)	0.807	−0.03	29/42	5/18	2.75 (1)	0.097	−0.17
**Adolescence (T2)**																
Antibiotics (yes/no)	7/87	3/60	1/17	0.019 (1)	0.891	0.02	4/68	0/9	0.53 (1)	0.468	−0.08	6/65	1/22	0.42 (1)	0.515	−0.07
Hair sampling in winter (yes/no)	13/81	5/58	4/14	2.80 (1)	0.094	0.19	6/66	3/6	4.95 (1)	0.026*	0.249	34/37	15/8	2.09 (1)	0.148	−0.15

*Notes*: EtG: ethyl glucuronide in ng/g, cut-off-values at 10 ng/g (EtG_10_) and 154 ng/g (EtG_154_). HCC: hair cortisol concentration. HCC/P T1: HCC to hair protein ratio in pg/mg; ln-transformed; ln-transformed and z-standardized. HCC/W T2: HCC to hair weight ratio in pg/mg; ln-transformed; ln-transformed and z-standardized. APGAR score: average of three scores, best adaption = 10. BMI: child’s current body-mass-index in percentiles. SES: socioeconomic status, combination of maternal/paternal education level (4-level: < 9, 9, 10 or 13 years) and net family income (6-level: < 1000€ to > 5000€), sum-index, theoretical range: 3–14. Maternal psychopathology: global severity index (GSI) of the Brief Symptom Inventory (BSI), range 0–30. Hair sampling in winter: sampling in December, January or February. Different sample size due to missing data: APGAR: *n* = 92, Maternal psychopathology: T1: *n* = 93, T2: *n* = 92; BMI (T2): *n* = 93, SES (T2): *n* = 87. *p < .05; **p < .01

**Table 2:** Sample characteristics and mean/frequency comparison for the risk factor prenatal maternal depressive symptoms

Parameters	Total *n* = 94	EPDS *n* = 93				
		EPDS < 10 *n* = 69	EPDS ≥ 10 *n* = 24			
	M (SD)	M (SD)	M (SD)	t (df)	*p*	d
**HCC/P T1 (pg/mg)**	164819.45 (498689.9)	108766.75 (168183.29)	329422.5 (940189.27)			
ln-transformed	10.97 (1.32)	10.821 (1.252)	11.374 (1.472)			
ln-transformed and z-standardized	0.022 (0.967)	−0.085 (0.918)	0.321 (1.08)	−1.78 (91)	0.078	−0.42
**HCC/W T2 (pg/mg)**	2.26 (1.522)	2.195 (1.519)	2.523 (1.541)			
ln-transformed	0.603 (0.682)	0.569 (0.689)	0.736 (0.653)			
ln-transformed and z-standardized	−0.004 (0.921)	−0.051 (0.931)	0.176 (0.883)	−1.04 (91)	0.301	−0.25
**birth outcomes**						
birth weight (grams)	3486 (490)	3478 (452)	3533 (589)	−0.47 (91)	0.637	−0.11
APGAR score	9.43 (0.56)	9.53 (0.45)	9.13 (0.73)	2.57 (29.53)	0.015*	0.76
**primary school age (T1)**						
age (years)	7.74 (0.81)	7.51(0.66)	8.36 (0.86)	−4.43 (32.79)	< 0.001**	−1.20
BMI (percentile)	51.90 (23.41)	52.01 (22.71)	50.41 (25.61)	0.29 (91)	0.775	0.07
SES index	11.35 (2.18)	11.45 (2.13)	11.0 (2.36)	0.87 (91)	0.389	0.21
maternal psychopathology	46.52 (13.77)	45.57 (13.22)	49.54 (15.31)	−1.21 (90)	0.228	−0.29
**adolescence (T2)**						
age (years)	13.28 (0.30)	13.26 (0.28)	13.34 (0.34)	−1.14 (91)	0.259	−0.27
BMI (percentile)	50.44 (29.83)	50.18 (28.97)	49.46 (32.23)	0.10 (90)	0.919	0.02
SES index	11.91 (2.03)	11.90 (2.09)	11.83 (1.9)	0.16 (84)	0.874	0.04
maternal psychopathology	45.89 (11.69)	44.41 (11.09)	50.17 (12.85)	−2.07 (89)	0.041*	−0.50
	***n***	***n***	***n***	***Χ***^***2***^ ***(df)***	***p***	***φ***
sex assigned at birth (female/male)	48/46	32/37	15/9	1.85 (1)	0.174	0.14
**primary school age (T1)**						
antibiotics (yes/no)	16/78	14/55	2/22	1.79 (1)	0.181	−0.14
hair sampling in winter season (yes/no)	34/60	27/42	7/17	0.76 (1)	0.383	−0.09
**adolescence (T2)**						
antibiotics (yes/no)	7/87	4/65	7/86	1.15 (1)	0.284	0.11
hair sampling in winter (yes/no)	13/81	8/60	5/19	1.20 (1)	0.273	0.11

*Notes*: EPDS: Edinburgh Postnatal Depression Scale. HCC: hair cortisol concentration. HCC T1: HCC/P ratio (HCC to hair protein ratio in pg/mg), ln-transformed and z-standardized. HCC T2: HCC/W ratio (HCC to hair weight ratio in pg/mg), ln-transformed and z-standardized. APGAR score: average of three scores, best adaption = 10. BMI: child’s current body-mass-index in percentiles. SES: socioeconomic status, combination of maternal/paternal education level (4-level: < 9, 9, 10 or 13 years) and net family income (6-level: < 1000€ to > 5000€), sum-index, theoretical range: 3–14. Maternal psychopathology: global severity index (GSI) of the Brief Symptom Inventory (BSI), range 0–30. Hair sampling in winter: sampling in December, January or February. Different sample size due to missing data: APGAR: *n* = 91, maternal psychopathology (T1 and T2): *n* = 92, BMI (T2): *n* = 92, SES (T2): *n* = 86. *p < .05; **p < .01

## Results

### Descriptive data and pre-analyses

Descriptive statistics of the sample’s characteristics (total and mean/frequency comparisons for each risk factor) are presented in Tables 1 and 2. The sample comprised 46 boys (48.9%) and 48 girls (51.1%). Adolescents’ school levels (assessed as intended total years of school attendance) ranged from low (≤ 9 years, 6.4%) to medium (≥ 10 years, 30.8%) to high (12–13 years, 62.8%). The majority of mothers were of high educational level (58.5% university entrance qualification). A valid maternal prenatal EPDS value was available for *n* = 93 (98.9%) participants, a valid meconium EtG value for *n* = 81 (86.2%) children and the mother’s self-evaluation report of prenatal alcohol consumption for all participants. *N* = 18 participants (22.2%) had EtG values ≥ 10 ng/g (EtG_10_+) and for *n* = 9 (11.1%), EtG values were ≥ 154 ng/g (EtG_154_+). *N* = 23 (24.5%) of the participating women confirmed consuming alcohol during pregnancy in their self-report (self-report+), most of them (*n* = 22) stated that they drank rarely during pregnancy. The association between self-report and EtG status was not significant for EtG_10_+ (*χ*^*2*^ = 3.72, *φ* = 0.21, *p* = .054) and EtG_154_+ (*χ*^*2*^ = 0.00, *φ* = 0.00, *p* = 1.00), either. Regarding prenatal maternal depressive symptoms, *n* = 24 (25.5%) mothers had EPDS scores ≥ 10. Of these, *n* = 3 also were assigned to the EtG_10_+ group, *n* = 2 to the EtG_154_+ group.

The following significant differences between risk group-positive and -negative children were identified (Tables 1 and 2): EtG cutoff 10 ng/g: BMI at T1; EtG cutoff 154 ng/g: maternal psychopathology at T1, hair sampling in winter at T2; EPDS: APGAR score, age at T1, maternal psychopathology at T2. To identify possible confounding variables with regard to the HCC, correlative analyses and mean comparisons (*t*-tests) were performed (Table 3). At T1, significant associations with the HCC were found for the following variables: birth weight, age at T1 and hair sampling in winter. At T2, only hair sampling in winter was significantly - and inversely to the T1 HCC correlations - associated with the HCC (Table 3). The variables used as covariates per ANCOVA are indicated in Table 4.

**Table 3:** Correlations and mean comparisons between the HCC/P-ratio (T1) / HCC/W-ratio (T2) and potential confounders

Potential confounders	HCC at T1				HCC at T2			
	*r*		*p*		*r*		*p*	
birth weight	−0.23		0.026*		−0.04		0.704	
APGAR score	−0.20		0.052		0.04		0.690	
age^a^	0.44		< 0.001**		0.05		0.625	
BMI (percentile)^a^	−0.00		0.938		−0.14		0.187	
SES^a^	−0.05		0.640		−0.10		0.385	
maternal psychopathology^a^	−0.10		0.358		−0.08		0.423	
	***t***	***p***	***df***	***d***	***t***	***p***	***df***	***d***
sex assigned at birth	1.74	0.085	81.77	0.36	−0.66	0.511	92	−0.14
use of antibiotics^a^	0.54	0.588	92	0.15	−0.16	0.877	92	−0.06
hair sampling in winter^a^	3.11	0.002**	89.36	0.60	2.13	0.028*	91	−0.69

*Notes*: a: assessed at T1 or T2, respectively. HCC: hair cortisol concentration. HCC T1: HCC/P ratio (HCC to hair protein ratio in pg/mg), ln-transformed and z-standardized. HCC T2: HCC/W ratio (HCC to hair weight ratio in pg/mg), ln-transformed and z-standardized. APGAR score: average of three scores, best adaption = 10. BMI: child’s current body-mass-index in percentiles. SES: socioeconomic status, combination of maternal/paternal education level (4-level: < 9, 9, 10 or 13 years) and net family income (6-level: < 1000€ to > 5000€), sum-index, theoretical range: 3–14. Maternal psychopathology: global severity index (GSI) of the Brief Symptom Inventory (BSI), range 0–30. Hair sampling in winter season: sampling in December, January or February. Different sample size due to missing data: APGAR: *n* = 92, Maternal psychopathology: T1: *n* = 93, T2: *n* = 92; BMI (T2): *n* = 93, SES (T2): *n* = 87. *p < .05; **p < .01

### Risk group differences and longitudinal outcomes for HCC

In all ANCOVAs, the following variables were controlled for: birth weight, children’s age T1, hair sampling in winter at T1, hair sampling in winter at T2. Additionally, maternal psychopathology was used as covariate in the ANCOVAs for the groups EtG_154_ (maternal psychopathology at T1) and EPDS (maternal psychopathology at T2).

#### PAE

**Fig. 1:**
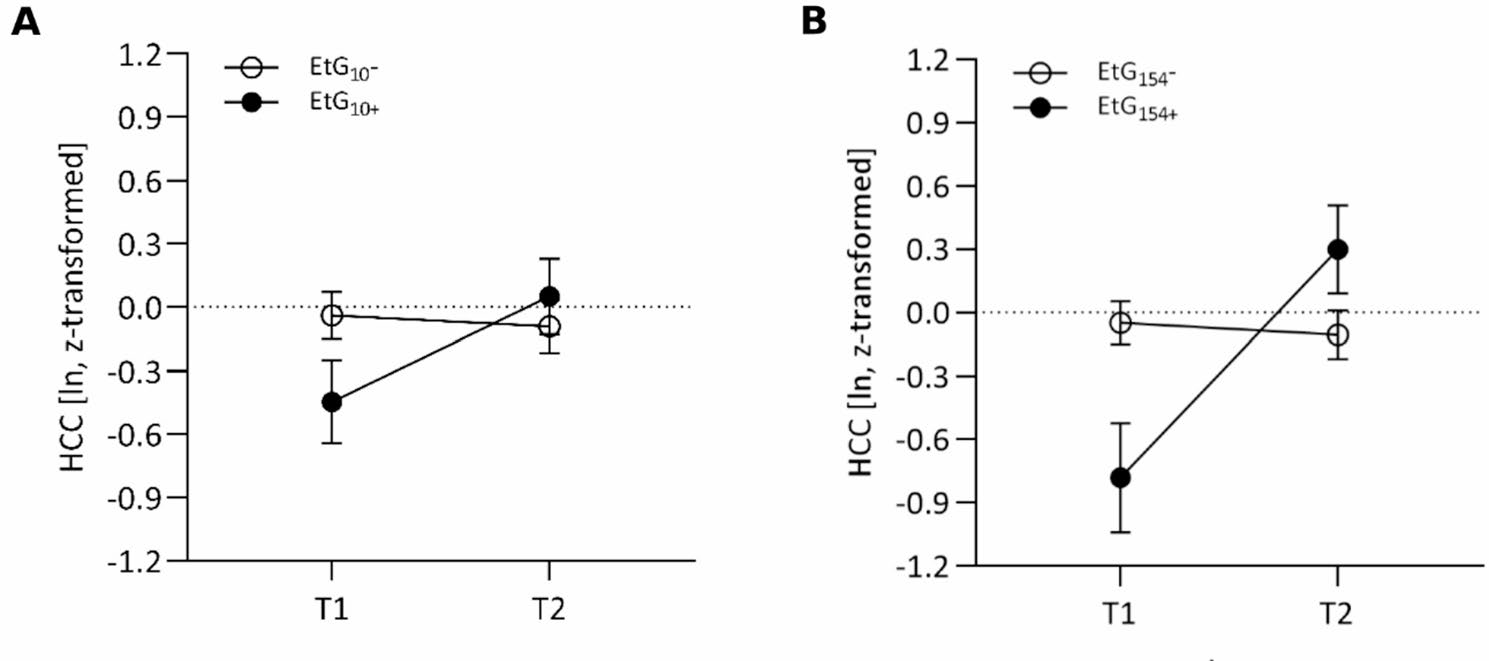
Mean HCC levels at T1 and T2 of the EtG risk groups, **(A)** EtG10, **(B)** EtG154, compared to the respective control group. Data are presented as mean +/- SEM

For the EtG_10_ group, there was no main group effect, but a significant effect of time (*p* = .033, *η*_*p*_^*2*^ = 0.059), as well as a statistically significant interaction effect (group × time) at small size (*p* = .045, *η*_*p*_^*2*^ = 0.053). At T1, EtG_10_+ HCC levels were non-significantly lower than EtG_10_ − HCC levels at T1 (*p* = .061). EtG_10_+ HCC levels increased significantly from T1 to T2 (*p* = .043), while the HCC levels of the EtG_10_− group remained stable over time (Tables 1 and 4; Fig. 1A) so that the difference between risk groups was attenuated at T2.

When considering the cut-off value EtG ≥ 154 ng/g, there was also a significant effect of time (*p* = .02, *η*_*p*_^*2*^ = 0.072) as well as a statistically significant interaction effect (group × time) at medium effect size (*p* = .01, *η*_*p*_^*2*^ = 0.088). *Post-hoc* tests showed that EtG_154_+ HCC levels were significantly lower than EtG_154_− HCC levels at T1 (*p* = .032) and increased significantly from T1 to T2 (*p* = .009), while the HCC levels of the EtG_154_− group remained stable over time. Accordingly, there was no significant difference between both groups at T2 anymore (Tables 1 and 4; Fig. 1B).

Regarding PAE assessed via self-reports, there was a significant effect of time at small effect size (*p* = .043, *η*_*p*_^*2*^ = 0.046), but no significant main effect of group, nor a significant interaction effect (group × time) (Table 4).

#### Prenatal depressive symptoms

Investigating the risk group defined by the factor ‘prenatal maternal depressive symptoms’, there was a significant effect of time at small effect size (*p* = .027, *η*_*p*_^*2*^ = 0.057), but no significant main effect of group, nor a significant interaction effect (group × time) (Tables 2 and 4).

**Table 4:** Repeated measures ANCOVAs for each risk group (within-subject factor: HCC at T1 and T2)

	F (df, df)	*p*	η_*p*_^2^
PAE: EtG_10_			
Time	4.701 (1, 75)	0.033*	0.059
Group	0.210 (1, 75)	0.648	0.003
Time × group	4.162 (1, 75)	0.045*	0.053
PAE: EtG_154_			
Time	5.653 (1, 73)	0.020*	0.072
Group	0.057 (1, 73)	0.811	0.001
Time × group	7.052 (1, 73)	0.010*	0.088
PAE: self-report			
Time	4.235 (1, 88)	0.043*	0.046
Group	0.731 (1, 88)	0.395	0.008
Time × group	0.076 (1, 88)	0.783	0.001
Prenatal depressive symptoms (EPDS)			
Time	5.100 (1, 84)	0.027*	0.057
Group	0.943 (1, 84)	0.334	0.011
Time × group	1.789 (1, 85)	0.185	0.021

*Notes*: Covariates: birth weight, age T1, hair sampling in winter T1, hair sampling in winter T2. Additional covariates: PAE: EtG_154_: maternal psychopatholgy T1; EPDS: maternal psychopathology T2. EtG: ethyl glucuronide in ng/g, cut-off-values at 10 ng/g (EtG_10_) and 154 ng/g (EtG_154_). self-report: maternal self-report on prenatal alcohol consumption, assessed during third trimester of pregnancy. EPDS: Edinburgh Postnatal Depression Scale, completed during third trimester of pregnancy. *p < .05; **p < .01

### Functional relevance of HCC results

To investigate the functional relevance of the HCC results, partial correlations between the HCC and child’s emotional and behavioral problems were calculated for T1 and T2, controlling for potential confounders identified in the pre-analyses (Table 3). Table 5 displays the results for the total sample and for the risk groups that showed significant differences in HCC compared to their respective control groups.

In the total sample, there was no association found between HCC and child psychopathology during elementary school or adolescence. In the EtG_10_+ group, the emotional symptoms score at T2 was significantly higher than in the control group (mean EtG_10_+ = 3.5, *SD* = 2.87, mean EtG_10_− = 1.44, *SD* = 1.65; *t* = -2.900, *df* = 20.317, *p* = .004). In the EtG_154_+ group, the emotional symptoms score at T1 was significantly higher than in the control group (mean EtG_154_+ = 3.11, *SD* = 2.15, mean EtG_154_− = 1.63, *SD* = 1.55; *t* = -2.592, *df* = 79, *p* = .011). However, no significant association was found between HCC and childhood psychopathology at T1 or T2 in the EtG ≥ 10 ng/g risk group or in the EtG ≥ 154 ng/g risk group (Table 5).

**Table 5:** Partial correlations of HCC at T1 and T2 with the respective emotional and behavioral problems scores, rated at T1 and T2

	Total difficulties score	Emotional symptoms	Conduct problems
	*r*_*p*_ (*p*)	*r*_*p*_ (*p*)	*r*_*p*_ (*p*)
**Total sample**			
HCC (T1)	− 0.100 (0.344)	− 0.028 (0.789)	− 0.13 (0.212)
HCC (T2)	0.161 (0.127)	0.160 (0.129)	0.106 (0.319)
**PAE: EtG**_**10**_**+** (*n* = 18)			
HCC (T1)	− 0.091 (0.746)	− 0.182 (0.517)	− 0.151 (0.591)
HCC (T2)	0.035 (0.894)	0.265 (0.305)	− 0.167 (0.521)
**PAE: EtG**_**154**_**+** (*n* = 9)			
HCC (T1)	− 0.214 (0.684)	− 0.452 (0.368)	− 0.385 (0.452)
HCC (T2)	− 0.325 (0.432)	0.113 (0.790)	− 0.352 (0.392)

*Notes*: Total Difficulties score, Emotional symptoms and Conduct problems: German version of the Strengths and Difficulties Questionnaire (SDQ-Deu), rated by the mother at T1 and T2. Covariates: T1: birth weight, age T1, hair sampling in winter T1; T2: hair sampling in winter T2. HCC: hair cortisol concentration. HCC T1: HCC/P ratio (HCC to hair protein ratio in pg/mg), ln-transformed and z-standardized. HCC T2: HCC/W ratio (HCC to hair weight ratio in pg/mg), ln-transformed and z-standardized. EtG_10_+: ethyl glucuronide values ≥ 10 ng/g. EtG_154_+: ethyl glucuronide values ≥ 154 ng/g

## Discussion

The aim of the present study was to determine the long-term effects of the prenatal risk factors alcohol exposure and maternal depressive symptoms on the child’s cortisol stress system, assessed via cumulative hair cortisol. It complements previous research in that the two developmental time points primary school age (six to nine years) and early adolescence (twelve to 14 years) were assessed to investigate long-term effects of the prenatal risk factors on hair cortisol concentrations in affected children and adolescents.

We were able to show that children with a positive EtG result in meconium displayed reduced HCC at primary school age compared to unaffected peers - a difference that leveled off in adolescence. In contrast, children whose mothers reported depressive symptoms during pregnancy did not exhibit altered HCC in primary school and adolescence compared to an unaffected control group.

Regarding the biomarker meconium EtG, the differences in HCC at T1 observed when using the cut-off value EtG ≥ 10 ng/g turned into a significant PAE-associated effect with the EtG threshold ≥ 154 ng/g, which can be interpreted as a more severe alcohol exposure [5]. The observed differences between affected and unaffected children and adolescents did not remain constant in terms of direction and extent; rather, the long-term effect of the prenatal risk factor alcohol was reflected in a distinct change in the cortisol secretion of those affected compared to those unaffected over time: While the cortisol concentrations of the children unaffected by prenatal alcohol exposure remained almost constant from primary school age to early adolescence, alcohol-exposed individuals displayed a significant hypocortisolism at T1, which is in line with our previous findings [36]. Interestingly, this difference was not observed at T2 anymore. Of course, it is important to consider the limited sample size in the EtG_154_+ group when interpreting these results, which is further discussed in the limitations section. Previous research has indicated that PAE can result in both reduced (hypocortisolism) and increased cortisol release (hypercortisolism) in the offspring [21, 51]. Keiver et al. [52] found hypercortisolism in PAE-affected children and adolescents between the ages of six and 14, with significantly higher salivary cortisol concentrations in the afternoon and before bedtime compared to the control group. McLachlan et al. [53] observed higher basal cortisol concentrations in saliva in the evening, lower, though not significantly so, cortisol levels in the morning and a flatter curve of basal cortisol secretion during the day in children and adolescents with PAE and early adverse life experiences.

Our observation that the effects of PAE on the cortisol stress system appear to be lessened in adolescence may be due to the fact that, in addition to the prenatal period and early childhood, the pubertal developmental stage is also a sensitive time window for regulating the HPA axis [21]. For instance, higher basal cortisol concentrations and greater cortisol reactivity have been found with increasing age and advancing pubertal status (e.g., [54, 55]). Koss and Gunnar [21] further suggest that the activity of the HPA axis during puberty may be subject to greater inter-individual variability. Quevedo et al. [56] found a flattened cortisol awakening response (CAR) curve in formerly institutionalized adopted adolescents in early puberty, whereas no effect on their cortisol stress system was found in adolescents whose puberty was already more advanced (mid or later pubertal status). From this, the authors concluded that puberty may represent a window of recalibration of the HPA axis. Animal models indicate that positive social experiences during puberty can correct the unfavorable influence of early risks [57]. We therefore suggest that the PAE-associated HPA axis dysregulation found at primary school age in our small sample leveled off in early adolescence - whether due to pubertal development per se and/or additional influences in the meantime.

Consistent with our prior research [5, 36], the findings of the present study support the superior validity of the biomarker EtG over maternal self-report of prenatal alcohol consumption in predicting later child developmental outcomes, specifically the cortisol stress system: While differences in HCC were identifiable between alcohol-exposed and non-exposed children and adolescents based on their EtG levels (EtG-positive vs. EtG-negative), no such group differences were observed based on maternal self-reports. The fact that statistically significant group differences were only detected when applying higher EtG threshold values (EtG ≥ 154 ng/g) in our study raises the question to which extent moderate or low alcohol consumption during pregnancy affect the development of the child’s HPA axis. First, it is important to note that the difference observed in the EtG10-group at T1 was not statistically significant, but there was a significant interaction of group and time. Furthermore, the present data was obtained from a sample of the general population, rather than a clinical sample. None of our underage participants had any visible impairments or a diagnosis of FASD. At the same time, we were not able to determine the exact amount of alcohol a mother consumed during pregnancy, as there is no ethical way to accurately measure the correlation between the amount of alcohol consumed and the resulting EtG value in the child’s meconium [37]. It is also important to note that lower EtG thresholds have revealed PAE-associated effects on mechanisms not covered by the HCC. For example, Eichler et al. [5], investigating the same cohort as the present study, observed changes in event-related potentials in children with an EtG cut-off value ≥ 10 ng/g compared to the control group. Likewise, also using the same cohort in each case, Frey et al. [58] reported epigenetic changes in affected children of primary school age with an EtG cut-off value ≥ 30 ng/g and Maschke et al. [59] found changes in inflammatory markers (high-sensitivity C-reactive protein, hsCRP) in the blood of prenatally alcohol-exposed adolescents. Overall, findings from both animal models and human studies indicate that there is no safe limit for alcohol consumption during pregnancy [60]. A meta-analysis of 34 published cohort studies showed a link between low and medium levels of PAE and behavioral problems during childhood [61]. In addition, studies indicate that there are individual differences, such as genetic factors, that may influence the susceptibility of both the mother and child to the teratogenic effects of PAE [60].

Although we could not determine the amount of alcohol consumed that actually reached the child and caused the EtG concentration, our results do suggest that there are differences in HPA axis activity between PAE-affected and unaffected children and adolescents. The clinical significance of these findings is that a dysregulation of the HPA axis is known to have long-term effects on both physical and mental health. Both hypercortisolism and hypocortisolism are associated with an increased risk of cardiovascular disease, obesity, Cushing’s disease, mood disorders, and anxiety disorders [62, 63]. The present results suggest that there may be developmental protective factors that support normalization of hair cortisol levels in the EtG risk group from primary school to early adolescence. The precise nature and mechanisms of action of such protective factors are still the subject of future research. In a further study on advancing adolescence, it should be investigated whether the statistical levelling of the risk factor PAE with regard to HCC persists, or whether there is a shift in towards HPA axis dysregulation (hypo- to hypercortisolism) in middle or late adolescence, which could not yet be observed in early adolescence. Similarly, it is important to verify the present results in the context of larger samples within the risk constellations.

Regarding the second prenatal risk factor investigated in our study, prenatal maternal depressive symptoms were not associated with the cumulative cortisol release compared to the control group. Most research on this has focused on short-term HPA axis activity, such as basal cortisol activity or cortisol reactivity, measured in saliva or blood (e.g., [25–28, 33, 64]). However, the nature of HPA axis dysregulation observed in these studies varies: Some have reported a link between prenatal depression and overactivity of the infant cortisol stress system [25, 26, 33], whereas others have observed hypocortisolism in the affected offspring [27, 28, 64]. Fewer studies have examined the association between prenatal symptoms of depression and markers of cumulative cortisol release (e.g., [31, 34, 35, 65]). In contrast to our findings, Karl et al. [65] report that higher prenatal depressive symptoms showed a significant association with higher infants’ neonatal hair cortisol. Nevertheless, our results are in line with those of other previous studies that could not identify an association between maternal psychopathological symptoms and offspring hair cortisol levels [31, 34, 35] - although [35] and [34] do report a positive association with hair cortisone. Of course, it cannot be ruled out that these discrepancies might be due to methodological differences (in, e.g., cm of hair examined / cortisol extraction and assaying method / EPDS score threshold etc. [31]). Yet overall, our results support the assumption that exposure to maternal psychopathology and stress during intrauterine development only affects immediate basal cortisol release, not cumulative release [34].

Evidence suggests that dysregulation of the cortisol stress system is associated with a range of mental impairments, such as depressive symptoms, or anxiety [16, 18]. The present study investigated the association between altered HCC in PAE-affected offspring and psychopathological abnormalities in early adolescence. The fact that no significant associations were found within the EtG risk groups warrants future studies with larger samples sizes to further investigate this issue.

### Limitations

The biomarker meconium-EtG does not allow any conclusions to be drawn about the mother’s actual alcohol consumption during pregnancy. As meconium EtG is a relatively new biomarker, there are still too few comparative data to allow a valid interpretation of EtG values and, for example, to define uniform cut-off values for the extent of prenatal alcohol exposure (e.g. low, medium, high alcohol consumption). In addition, the biomarker EtG does not provide information on the ‘pure’ alcohol consumption of the mother during pregnancy, as it may be altered by other factors (e.g. fruit juices, mouthwash). The women participating in the FRAMES project were not asked about the exact time, drinking pattern or exact amount of alcohol they consumed. Additional recording of these aspects would be highly recommended for future studies. Future studies should also continue to focus on the individual response of the child to alcohol exposure during pregnancy, as previous research suggests that the same amount of maternal alcohol consumption does not result in the same impairment or the same amount of EtG in meconium in all children [37, 60]. Still, it should be emphasized that in the present study, in contrast to maternal self-report of alcohol consumption during pregnancy, meconium EtG was associated with HCC as a relevant outcome. Accordingly, this biomarker has significant potential in clinical practice, not least because of its higher validity compared to self-reported PAE [66] and the relatively simple and non-invasive possibility to collect meconium from newborns.

In the present study, prenatal depressive symptoms of pregnant women were assessed during the third trimester of pregnancy using the widely validated [8] EPDS self-report instrument. The presence of postpartum depression in the mother was not assessed, although this is also considered a significant risk factor for the child’s physical, cognitive, and emotional development [67]. At the same time, it is likely that many of the participating mothers who were depressed after childbirth had depressive symptoms prior to the birth of their child, as prenatal depressive symptoms are considered the strongest predictor of postnatal depression [11]. Similarly, in non-psychiatric samples such as the present one, depression scores are often higher during the prenatal period than during the postpartum episode [67]. However, the mothers’ current psychopathological symptoms were assessed using the BSI at T1 and T2. In the present study, women were asked about their depressive experiences only once during the third trimester of pregnancy. Considering that the first and third trimesters of pregnancy, in particular, appear to be sensitive time windows with regard to long-term changes in the HPA axis [21], repeated assessment of expectant mothers’ depressive symptoms during the course of pregnancy would be recommended for future work.

Regarding the operationalization of the HCC, it is important to note that, due to technical reasons, the cortisol extraction method and reference values used to normalize the HCC differed between the two measurement times (HCC/W ratio at T1 and HCC/P ratio at T2), making it difficult to compare the results. Although the HCC/W ratio and the HCC/P ratio of the same sample have been reported to show a high correlation [42], using different ratios at the two measurement times may have affected the longitudinal associations. However, to partially account for this difficulty in comparability, *z*-transformations were performed on the respective HCC ratios. Furthermore, a potential impact of sample storage time on hair cortisol concentrations cannot be excluded. Berger et al. propose that hair can be stored for at least five years without compromising HCC [68], whereas a more recent study reports that hair cortisol declines within one year of storage [69]. Consequently, sample storage time and conditions should be considered as potential confounding factors in future studies.

Final critical points to note are that no adjustments to multiple testing were made and that the sample size is small, particularly within the EtG_154_+ group (*n* = 9). Therefore, caution must be taken when interpreting the present results regarding an association between PAE and the cortisol stress system in childhood and adolescence. Future studies should aim to increase the sample size in order to improve the reliability of the effects and to be able to examine moderating factors, such as the child’s sex.

## Conclusion

The present study provides further evidence of long-term effects of alcohol consumption during pregnancy on the HPA axis development of the child. These effects are thought to have lasting implications for the child’s stress processing and neurobehavioral development. Accordingly, the present findings emphasize the significance of preventing avoidable risks during pregnancy, such as alcohol consumption, which would significantly enhance the developmental prospects of the unborn child. However, subsequent studies with larger sample sizes are required to validate our findings and elucidate the PAE-associated development of the HPA axis during the progression of adolescence. Additionally, the issue of whether there is an association between the risk factor prenatal depressive symptoms and the child´s HPA axis, warrants further study.

## Data Availability

The data that support the findings of this study are not publicly available due to their containing information that could compromise the privacy of research participants, but are available from A.E. on reasonable request.
